# Physicochemical and Instrumental Flavor Analysis of Plant-Based Drinks with Plant Powder Additions

**DOI:** 10.3390/foods14152593

**Published:** 2025-07-24

**Authors:** Joanna Kolniak-Ostek, Agnieszka Kita, Davide Giacalone, Laura Vázquez-Araújo, Luis Noguera-Artiaga, Jessica Brzezowska, Anna Michalska-Ciechanowska

**Affiliations:** 1 Department of Fruit, Vegetable and Plant Nutraceutical Technology, Faculty of Biotechnology and Food Science, Wrocław University of Environmental and Life Sciences, 37 Chełmońskiego Str., 51-630 Wroclaw, Poland; joanna.kolniak-ostek@upwr.edu.pl (J.K.-O.); jessica.brzezowska@upwr.edu.pl (J.B.); 2 Department of Food Storage and Technology, Faculty of Biotechnology and Food Science, Wrocław University of Environmental and Life Sciences, 37 Chełmońskiego Str., 51-630 Wroclaw, Poland; agnieszka.kita@upwr.edu.pl; 3 Department of Green Technology, University of Southern Denmark, 5230 Odense, Denmark; dg@igt.sdu.dk; 4 Faculty of Gastronomic Sciences, Basque Culinary Center, Mondragon Unibertsitatea, 101 Paseo Juan Avelino Barriola, 20009 Donostia-San Sebastián, Spain; lvazquez@bculinary.com; 5 GOe Tech Center, Technology Center in Gastronomy, Basque Culinary Center, 101 Paseo Juan Avelino Barriola, 20009 Donostia-San Sebastián, Spain; 6 Centro de Investigación e Innovación Agroalimentaria y Agroambiental (CIAGRO-UMH), Miguel Hernandez University, 03312 Orihuela, Spain; lnoguera@umh.es

**Keywords:** powders, plant-based beverages, electronic nose, electronic tongue, polyphenols

## Abstract

This study explored the use of fruit- and herb-based powders as fortifying agents in soy- and oat-based beverages. Developed using a New Product Development approach, the powders were derived from underutilized plants rich in bioactives but with limited sensory appeal. Formulations included powders from both widely available fruits, such as apple and pear, chosen for their accessibility and economic relevance, and less commonly consumed fruits, such as Japanese quince, rosehip, and rhubarb, which are often discarded due to sour or astringent flavors. Processing these into powders helped mask undesirable sensory traits and enabled incorporation into beverage matrices. Physicochemical analyses confirmed their technological suitability, while high polyphenol content indicated potential health benefits. Importantly, no process contaminants (furfural, 5-hydroxymethyl-*L*-furfural, and acrylamide) were detected, supporting the powders’ safety for food use. The integrated application of an electronic tongue and nose enabled objective profiling of taste and aroma. The electronic tongue distinguished taste profiles across formulations, revealing matrix-dependent effects and interactions, particularly with trehalose, that influenced sweetness and bitterness. The electronic nose provided consistent aroma differentiation. Overall, the results highlight the potential of these underutilized plant powders as multifunctional ingredients in plant-based beverage development. They support product innovation aligned with consumer expectations for natural, health-promoting foods. Future work will include sensory validation with consumer panels.

## 1. Introduction

Fruits are a vital component of a healthy diet, providing a rich source of pectins, organic acids, polyphenols, and other health-beneficial compounds. Their appealing sensory qualities contribute to their widespread consumption, with apples and pears being among the most widely grown and economically accessible fruits [1]. Given their availability, popularity, and affordability, these fruits present an excellent foundation for developing innovative food products. However, large-scale production may lead to surpluses, resulting in economic losses for producers. In contrast, fruits and vegetables with less desirable sensory attributes, such as Japanese quince, rosehip, or rhubarb, which are characterized by intense sourness or astringency [2], are often underutilized despite their rich content of bioactives. Incorporating these fruits into the diet can enhance the intake of health-promoting nutrients and boost the composition of less nutritionally dense foods. The specific taste profiles of these products generally lead to their consumption in processed forms, such as additives to juices, desserts, or supplements [3]. Among advanced technologies, powdering offers effective solutions for processing both surplus and less appealing fruits and vegetables by masking their undesired taste and smell, making the resulting components easier to incorporate into other foods. When added to other products, the powders not only go unnoticed in flavor but also enrich the product with beneficial bioactive compounds that would otherwise not be naturally present.

Transforming highly perishable plants into powders extends their availability beyond their natural season, ensuring year-round market presence. Such processing also contributes to food loss reduction by increasing the shelf life of these raw materials [4]. Their stable form facilitates transportation and storage while minimizing spoilage. Furthermore, plant-based powders allow for the fortification of different products [5], such as bakery items, dairy products, and beverages [6].

Their versatile applications support the formulation of new food products with improved nutritional properties. However, powdering requires extensive technological knowledge, as the transformation of raw materials—whether in liquid or solid form—necessitates the development of appropriate formulations, as well as the adjustment of drying techniques and processing parameters, to obtain products in powder form. These processing methods influence the retention of bioactive compounds, affecting the final product’s potential health benefits. Among advanced drying techniques, spray drying is widely used due to its efficiency in producing nutrient-dense powders, with similar effects on the presence and content of bioactives as freeze-drying [7,8], making it suitable for thermolabile ingredients. Moreover, plant-based powders offer an excellent alternative to artificial additives by providing natural components, and at the same time can affect color, flavor, and nutritional enhancement [5,9]. Fortification with bioactive-dense powders can target different groups of daily consumed foods, and one of these could be plant-based beverages, such as oat or soy drinks [10], which may be perceived as less appealing due to potential sensory shortcomings, requiring reformulation [5]. The processing of plant raw materials with high sugar content may lead to undesirable transformations, including non-enzymatic browning and degradation of bioactive compounds [11]. Additionally, thermal treatments can contribute to the formation of process contaminants, potentially affecting both product safety and quality. Depending on the form and composition of raw materials (the presence and content of sugars and organic acids), carriers are commonly used for drying, and they have been found to influence the generation of Maillard reaction products [12]. One of the strategies to minimize this risk is the use of trehalose as a carrier in the production of fruit powders from liquid forms such as juices. As a non-reducing sugar, this carrier may help to prevent unwanted Maillard reactions and thermal degradation, improving the solubility and thus application potential of the final product [13]. Furthermore, the structure of powders can vary depending on their composition and the proportions of raw materials used in their production, which can affect their solubility. These differences could, in turn, affect the release of flavor compounds and therefore the sensory profile and perception of the final product. Their potential impact on the sensory perception of various matrices to which they are applied remains insufficiently understood. Moreover, current research on their application in food systems is limited, highlighting a significant knowledge gap in this area. Most available studies focus on liquid plant extracts or freeze-dried materials derived from whole fruits or their fragments, which are often insoluble in beverages and act mainly as particulate additives rather than fully integrating into the product matrix. Research on soluble plant powders, particularly those obtained from plant juices through spray drying, remains scarce, despite their potential as fully dissolvable ingredients in beverage formulations. In addition, the sensory profiling of such powders after drying and reconstitution, especially using advanced tools such as an electronic nose and electronic tongue, is underexplored.

Taking the above into consideration, it was hypothesized that fruits and vegetables with poor sensory appeal could be used to produce plant-based powders capable of enhancing sensory properties when added in adjusted amounts to different plant liquid matrices (soy and oat beverages). Furthermore, this study assumes that specific physicochemical properties, such as the solubility or polyphenol profiles of the spray-dried powders, may influence the sensory attributes of the final products. This targeted hypothesis aims to improve the focus and applicability of the findings in the context of plant-based beverage development.

Thus, the aim of this study was to assess the quality of plant powders in terms of physicochemical properties and to investigate their impact on the polyphenols content of plant-based beverages containing them. Using soy and oat beverages as distinct matrices, the study also examined how the composition and interactions of these powders influence flavor perception through electronic nose and tongue analyses. The findings will contribute to a deeper understanding of the potential of plant powders to enhance the functional and sensory profiles of plant-based beverages, supporting their application in future product development.

## 2. Materials and Methods

### 2.1. Raw Materials and Compositions Design for Drying

The materials used in this study consisted of apples, pears, rhubarb, Japanese quince, rosehip, and lemon balm, all sourced from the local market in Wrocław, Poland (June, 2024). Apple, pear, and rhubarb juices were made using a Sana Horeca EUJ-909 juicer (Sana Products Ltd., Ceske Budejovice, Czech Republic), followed by pasteurization in a Thermomix (Vorwerk SE & Co. KG, Wuppertal, Germany) at 90 °C for 10 min. Further, the juices were cooled to room temperature and used for the preparation of juice blends.

Freshly ground Japanese quince fruits (sourced from Leokadiów, Poland) were immediately subjected to hydraulic pressing (SRSE, Warsaw, Poland) to obtain the juice. Rosehip juice was prepared by enzymatic treatment of the fruit pulp with 1.5 mL of Pectinex^®^ SPL (Novozymes, Bagsværd, Denmark) per 1 kg of pulp for 24 h at ambient temperature, followed by hydraulic pressing. Lemon balm infusion was extracted by infusion of 4 g of dried herb in 100 mL of boiling water for 10 min.

The composition of the powders was developed through a two-step process: (1) idea generation and (2) concept development, following a consumer-driven approach focused on natural products and the use of locally sourced plant materials. The formulation concepts were based on combining two popular juice matrices, i.e., apple and pear juices, with less palatable but bioactive-rich ingredients, such as rhubarb, Japanese quince, and rosehip. Selected compositions were further enriched with lemon balm infusion to enhance their potential functional properties.

The selection of specific combinations was guided by feedback from a pre-experimental focus group conducted to support the development process. Both blends were developed through experimental design, aiming to incorporate at least 30% of the raw materials (juices) considered less appealing in terms of flavor but rich in bioactive compounds and nutritional value. These components were purposefully included to enhance the health-promoting properties of the final powders. The prepared blends were then subjected to drying and reconstitution tests. Ingredient proportions were adjusted to minimize the presence of off-flavors, which could result from the intrinsic taste of the bioactive-rich components and from potential interactions among the ingredients. The final powder compositions based on apple (Variant 1; V1) and pear (Variant 2; V2) are presented in Table 1.

To transform the liquid blends into powdered form, a spray drying technique was applied using an APV ANHYDRO LAB1 spray dryer (Østmarken, Søborg, Denmark). Trehalose, used as a carrier, was incorporated at 20% (*w*/*w*), which was determined through preliminary testing. The Brix values of V1 and V2 were 17.1 °Brix ± 0.0 and 15.6 °Brix ± 0.0, respectively. The process was carried out at an inlet temperature of 170 ± 2 °C, with an outlet temperature of 80 ± 5 °C. The liquid was fed at a rate of 3 L/h using a nozzle air pressure of 0.25 MPa. The drying was performed in two technical replicates (*n* = 2).

### 2.2. Application of Powders to Plant Beverages

Prior to the experiments, preliminary sensory evaluations were conducted to assess the compatibility of the powder additions with commonly available plant-based beverages, including soy, oat, almond, and coconut. Based on taste acceptability, soy (S) and oat (O) beverages were selected for further study, as the other options considered (almond and coconut) were rated less favorably.

To produce the enriched versions of the plant-based beverages, 2.4 g of the selected powder (V1, V2) were dispersed in 100 mL of commercially purchased oat drink (O), i.e., O(V1) or O(V2), or soy drink (S), including S(V1) or S(V2) (no added sugar; Alpro, Danone). The concentration was experimentally established, considering the sensory performance of each powder within the context of the specific oat or soy beverage matrix. For the comparison, samples without powders were treated as controls, i.e., O(C) and S(C), as well as only trehalose-added beverages, i.e., O(T) and S(T), and beverages based on water and V1 and V2 products, i.e., W(V1) and W(V2). The mixtures were manually agitated until full dissolution was achieved. All beverage samples were freshly made on the day of analysis.

### 2.3. Product Characterizations

#### 2.3.1. Moisture Content

The moisture content of powders was determined according to Figiel [14] using a vacuum oven at 80 °C for 24 h (*n* = 2).

#### 2.3.2. Water Activity (*a_w_*)

The *a_w_* was examined using the Water Activity meter (Dew Point, 4TE, AQUA LAB, Pullman, WA, USA) at 25 °C (*n* = 2).

#### 2.3.3. Color

The color of the powders was assessed in triplicate (*n* = 3) using a Minolta Chrome Meter CM-700d (Konica Minolta, Inc., Osaka, Japan) based on the CIEL*a*b* color system.

#### 2.3.4. Water Solubility Index (WSI)

The WSI was determined following the procedure described by Vicente et al. [15] with minor modifications. Exactly 0.5 g of the powder (dry mass) was mixed with 5 mL of water, vortexed for 60 s, and then allowed to stand at room temperature for 10 min. After another 60 s of vortexing, the mixture was centrifuged for 25 min at 3000× *g*. The supernatant was removed and transferred to a pre-weighed Petri dish. The dish was then dried at 110 °C overnight, and the weight of the remaining solids was measured after the water had evaporated. The WSI was calculated as the amount of soluble solids in grams per 100 g of dry matter (*n* = 4).

#### 2.3.5. Electron Microscopy

The powder samples were mounted onto a metal stage using carbon tape and coated with a 5 nm gold layer (Leica EM ACE200; Leica Mikrosysteme GmbH, Vienna, Austria). Images were captured using a Phenom XL scanning electron microscope (Thermo Fisher Scientific, Waltham, MA, USA) with an accelerating voltage of 10 kV and a chamber pressure of 10 Pa. For each sample, at least six images were taken at magnifications of 200, 500, and 1000×.

#### 2.3.6. Process Contaminants

For the analysis of process contaminants, including furfural (FF) and 5-hydroxymethyl-*L*-furfural (HMF), approx. 100 mg of powder was weighted (*n* = 2), followed by the addition of 20 mL of sodium acetate buffer. Samples were vortexed for 2 min and centrifuged for 10 min at 8000 rpm. The supernatant was filtered through a nylon syringe filter (0.45 μm) into chromatographic vials. The determination of FF and HMF was performed at Shimadzu Prominence i LC-2050C equipped with Phenomenex HyperClone column (5 μm, BDS C_8_, 130 A LC, 150 × 4.6 mm, Torrance, CA, USA) with mobile phase flow 1 mL/min, column temperature set up to 25 °C, autosampler temperature adjusted to 10 °C, and injection volume 10 μL. The wavelengths used for FF and HMF determination were 276 nm and 284 nm, respectively. The limit of detection (LOD) was established at 10 μg/g. For the acrylamide (AA) analysis, 1 g of powder was extracted with 15 mL of acetonitrile, followed by vortexing (2 min) (*n* = 2). Samples were centrifuged (10 min), and supernatant was filtered through a nylon syringe filter (0.22 μm) into chromatographic vials. The AA analysis was performed at Shimadzu QP2010se equipped with Phenomenex ZB-WAX30 column (25 μm, 30 × 25 mm, Torrance, CA, USA) with mobile phase flow 0.91 mL/min, carrier gas: helium, injection volume 1 μL at 230 °C. The LOD was established at 250 μg/kg. Identification of process contaminants was based on standard compounds. All determinations were performed in duplicate (*n* = 2).

#### 2.3.7. Identification and Quantification of Polyphenolic Compounds

Powder samples (0.3 g) were extracted with 1 mL of water (*n* = 2) (20 min under sonication: 300 W, 40 kHz; Sonic 6D, Polsonic, Warsaw, Poland) with intermittent shaking. The slurry was subsequently centrifuged at 19,000× *g* for 10 min. Then, the supernatant was filtered using a hydrophilic PTFE 0.20 µm membrane (Millex Samplicity^TM^ filter, Merck, Darmstadt, Germany) for analysis.

Soy and oat drinks as well as powder-added beverages were prepared for analysis according to the method described by Galkhoo et al. [16], with slight modifications. Briefly, liquid samples (2 mL) were extracted with 8 mL of 80% methanol acidified with 1% HCl (*v/v*) and sonicated for 20 min (300 W, 40 kHz; Sonic 6D, Polsonic, Warsaw, Poland), with occasional shaking. The resulting solution was centrifuged at 19,000× *g* for 10 min, and supernatant was filtered through PTFE 0.20 µm membrane (Millex Samplicity^TM^ filter, Merck, Darmstadt, Germany).

The approach used to identify polyphenolic compounds was that of Chandran et al. [17]. An ACQUITY UPLC system with a PDA detector and a G2 Q-Tof mass detector (Waters, Manchester, UK), with an ESI source operating in negative mode, was used. Polyphenols were isolated using a BEH C_18_ UPLC column (1.7 μm, 2.1 × 100 mm, Waters) at 30 °C for 15 min. The mobile phase contained 0.1% formic acid (*v*/*v*) (solvent A) and 100% acetonitrile (solvent B) using a linear gradient sequence and maintaining a constant flow rate of 0.42 mL/min. PDA spectra were acquired at 2 nm steps over the entire wavelength range of 200–600 nm. Retention times and spectra were compared with applicable standards. Analysis used comprehensive scan data-dependent mass spectrometry from *m*/*z* 100 to 2500. Leucine-enkephalin (*m*/*z* 554.2615 Da) was used as reference material at a concentration of 500 pg/mL. Mass spectrometry parameters were as follows: capillary voltage 2500 V, cone voltage 30 V, source temperature 100 °C, desolvation temperature 300 °C, and nitrogen flow rate 300 L/h. All measurements were performed in duplicate (*n* = 2) and expressed as milligrams per gram of powder and grams per 100 mL of plant-based beverages.

#### 2.3.8. Electronic Nose Analysis

The analysis was conducted using an ‘electronic nose’ apparatus equipped with an HS-100 autosampler, a sensor array, and two columns operating in parallel: a non-polar column (MXT5: 5% diphenyl, 95% methylpolysiloxane, 10 m length, 180 μm diameter) and a medium-polar column (MXT1701: 14% cyano-propylphenyl, 86% methylpolysiloxane, 10 m length, 180 μm diameter). Sample preparation for analysis involved weighing 1 g of the sample into 20 mL glass vials. The vials, sealed with a silicone-PTFE septum cap, were placed on the autosampler tray of the HERACLES ‘electronic nose’ system (Alpha MOS, Toulouse, France). The design of the device also allows analysis of the aroma in the solutions. After incubating the samples, a portion of the gas phase was taken from the vials, injected into a gas chromatograph with two flame ionization detectors, and separated into volatile components responsible for the sample’s aroma. Calibration of the apparatus was performed prior to the analysis using a solution of alkanes (from *n*-hexane to *n*-hexadecane). The chromatographic analysis allowed for the determination of the aroma component content (%). Relationships between aroma-related parameters of the samples were established using principal component analysis (PCA), which was applied to identify correlations between different aroma profiles. Identification of aromatic compounds and their characteristics (aroma descriptors) was carried out using the Alphasoft 14.2 andAroChembase software from Alpha MOS, Toulouse, France. All analyses were performed in triplicate (*n* = 3).

#### 2.3.9. Electronic Tongue Analysis

The ‘electronic tongue’ apparatus used in the study was equipped with an Ag/AgCl electrode and seven potentiometric sensors with varied cross-sensitivity to specific substances. The samples, dissolved in the appropriate solvent (depending on the substance being analyzed), were placed in glass containers in the autosampler of the apparatus for analysis. Using AlphaSoft 14.2 software (Alpha MOS, Toulouse, France) and the Taste Screening tool, the intensity of tastes—sour (sensor SRS), salty (sensor STS), umami (sensor UMS), sweet (sensor SWS), bitter (sensor BRS), metallic (GPS), and hot spicy (sensor SPS)—was determined. The intensity of each taste attribute was evaluated on a scale from 0 to 12 arbitrary units (a.u.), with boundary markings ranging from undetectable to very intense. The PCA method was also applied to the taste data, helping to establish relationships between taste-related parameters of the samples. The measurements were performed in five replicates (*n* = 5).

### 2.4. Statistical Analysis

The data are presented as average ± standard deviation. All determinations were performed at least in duplicate (*n* = 2). To determine significant differences (*p* < 0.05) between variables, the data were statistically analyzed using one-way analysis of variance (ANOVA), followed by either the Fisher’s LSD (Least Significant Difference) or Duncan's post hoc tests (Statistica 13.1, StatSoft, Kraków, Poland).

## 3. Results and Discussion

### 3.1. Physical Properties of Powder Compositions

The moisture content of two selected powder compositions obtained after spray drying was, on average, 2.12% (Table 2) with water activity at the level of 0.1357, which is a level regarded as appropriate to guarantee microbiological stability in powder-based compositions [18].

Samples V1 and V2 showed slight differences in color coordinates. While the L* values were comparable, variations were observed in the a* and b* coordinates. The V1 showed a slightly more reddish hue, likely due to the higher content of rhubarb and rosehip juice. In the case of water solubility index, both powdered compositions were considered as highly soluble (above 90%). The difference was linked to the initial composition of raw materials.

### 3.2. Scanning Electron Microscopy (SEM)

**Figure 1:**
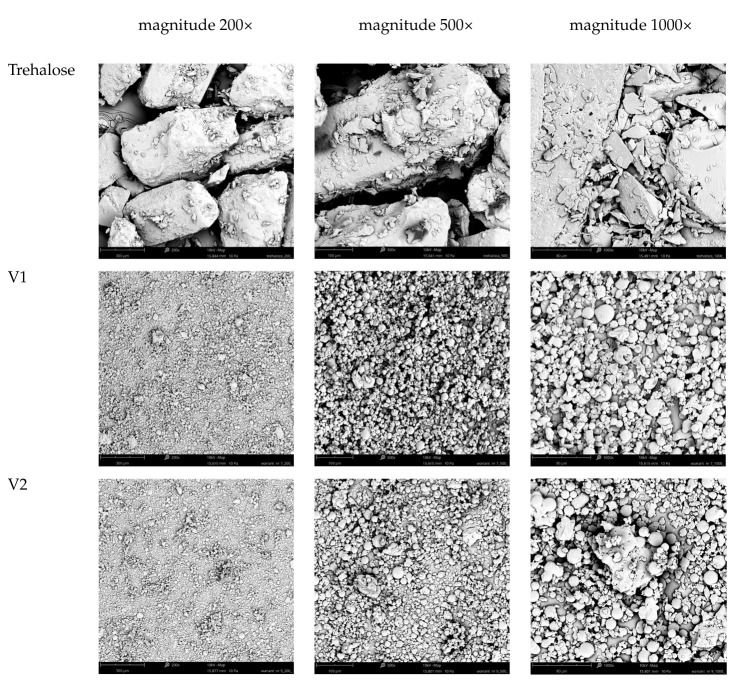
The SEM analysis of trehalose and plant-based powders produced with trehalose (V1 and V2) with magnifications of 200×, 500×, and 1000×.

The SEM images present a comparative analysis of three different powder samples: trehalose (used as a carrier) and two powders labeled as V1 and V2 (Figure 1). These samples were examined under different magnifications (200×, 500×, 1000×) to assess their morphological characteristics.

Trehalose (single carrier), visible in the first row, displayed a structure composed of large, irregular, and angular particles. These particles had sharp edges and fractured surfaces, indicative of a crystalline structure. Even at magnification up to 1000×, the trehalose particles remained coarse, with no evidence of finer or spherical features. Similarly to Khan et al. [19], it was observed that coarse trehalose particles were large and irregular in shape; however, following spray drying, the particles transformed into more spherical forms. Both V1 and V2 powders exhibited a different morphology. Already at 200× magnification, the powders appeared much finer and consisted of densely packed particles. At 500× magnification, their structures revealed numerous small, primarily spherical particles, which indicates that spray drying was a process applied to produce uniform, spherical, and smooth microparticles [20]. It can also be linked to the molecular structure of the carrier used for drying [21]. This trend continued at higher magnifications. For V1 powder, the images at 1000× magnification showed highly uniform, well-defined spherical microparticles, some of which exhibited surface texture, possibly indicating porosity or the presence of satellite particles. This structure suggested a refined and consistent formulation, likely contributing to favorable physical properties such as flowability or reconstitution behavior. The V2 powder, while also composed of small, spherical particles, appeared slightly less uniform than V1 at higher magnifications. Although the general morphology remained similar, with spherical shapes dominating the field, there might be slightly more particle aggregation or irregular surface features compared to V1. These subtle differences could be attributed to the differences in liquid feed composition, namely the juices used for its formulation (base for powder production), which can possibly affect performance characteristics, such as the solubility of powders. In summary, although V1 and V2 look quite similar, V1 appeared slightly more uniform and smoother in structure, which might suggest it possesses slightly better properties in terms of use or performance, like solubility (Table 2). It was indicated that powders with smoother surfaces exhibit improved flowability, faster and more uniform hydration, and lower viscosity, which facilitates easier processing and enhances mouthfeel [22]. Smaller particle size contributes to these effects by improving dispersibility [23]. Moreover, fine and smooth structures can enhance the release and bioavailability of health-promoting compounds such as phenolics and dietary fiber, increasing their nutritional value [24]. However, this improved functionality may come at the cost of reduced storage stability, highlighting the need for careful handling and formulation strategies.

### 3.3. Identification and Quantification of Polyphenolic Compounds

The analysis carried out using UPLC-PDA-Q/Tof-MS showed the presence of 67 polyphenolic compounds from the groups of phenolic acids, anthocyanins, flavan-3-ols, flavonols, and dihydrochalcones in the powders tested (Table 3).

In the V1 powder, the dominant compounds were phenolic acids, the content of which was at the level of 75.35 mg/g powder. In this group of compounds, the highest amount of caffeoylhexoside with [M − H]^−^ was determined at *m*/*z* 341.0627, which represented up to 59.68% of all phenolic acids. Such a high content of caffeoylhexoside results from its presence in all raw materials present in the powder compositions [25,26,27,28]. The second most abundant group was flavan-3-ols and procyanidins (20.84 mg/g), among which the dominant compound was (−)-epicatechin (37.33% of all flavan-3-ols). (−)-Epicatechin is a characteristic compound of apple and rhubarb [27,28], which constituted 81% of the mixture used to produce the powder. The analyzed powder also contained flavonols at 3.95 mg/g and dihydrochalcones, such as phloretin 2′-*O*-glucoside and phloretin 2′-*O*-xyloglucoside ([M − H]^−^ at *m*/*z* 435.0762 and 567.1042, respectively), which are apple-characteristic compounds, and 1.71 mg/g of rosehip and apple anthocyanins [25,28] were also determined.

In the V2 powder, the dominant group of compounds was revealed to be flavan-3-ols and procyanidins (62.91% of the total), the source of which was primarily pear juice, rosehip, and Japanese quince juice [25,29,30]. The dominant compound in this group was B-type procyanidin trimer with [M − H]^−^ at *m*/*z* 856.1913, the content of which was determined at the level of 22.14 mg/g of powder. Phenolic acids constituted 26.14% of the total polyphenols, and the dominant compound in this group was rosmarinic acid (4.92 mg/g), which was introduced to the recipe together with lemon balm infusion [26]. The tested powder was also characterized by the content of flavonols (9.52%), which are compounds present in all the raw materials used. Among the anthocyanins, constituting 1.42% of the mixture, cyanidin derivatives (glucoside and rutinoside with [M + H]^+^ at *m*/*z* 449.0984 and 595.1774, respectively) were determined, which were derived from rosehip juice [25].

The compositions of these powders, resulting from the use of different raw fruit materials and plant infusions, determined their specific phytochemical profiles. The analysis showed significant differences in the content and type of dominant compounds between the samples, which indicated potentially different characteristics, including antioxidant properties of both plant-based mixes. Taking these properties into account, powdered samples V1 and V2 were used to enrich plant-based beverages based on oat and soy. The aim of the fortification was to increase the content of polyphenols in the beverages and to investigate the effect of the addition of powders on the qualitative and quantitative composition of bioactive compounds. The results of the quantitative UPLC analysis of the content of polyphenolic compounds in plant-based beverages before and after the addition of fruit powders are presented in Table 4. In turn, Table S1 (Supplementary Materials) presents detailed characteristics of individual polyphenolic compounds determined in samples using the UPLC-PDA-Q/Tof-MS technique.

The oat drink contained 15.53 mg of polyphenolics per 100 mL of beverage. The dominant compounds were phenolic acids in the amount of 12.45 mg/100 mL and phytoalexins, like avenanthramide A and B, which are characteristic of oats [31], in the amount of 3.08 mg/100 mL (Table 4, Supplementary Table S1). The addition of fruit powders significantly increased the content of polyphenols in oat-based beverages. With the addition of V1 powder to the beverages, polyphenolic compounds from the group of anthocyanins, flavan-3-ols and procyanidins, flavonol, and dihydrochalcones were introduced, and the sum of the compounds increased to the level of 50.28 mg/100 mL. In the case of adding V2 powder, a twofold increase in polyphenol content was observed (to 36.19 mg/100 mL). Along with the powder, polyphenolic compounds from the anthocyanin group, flavan-3-ols, and flavonols were introduced into the plant beverage.

The soy drink was characterized by a higher content and diversity of polyphenolic compounds compared to the oat product. The total polyphenolic compound content in the soy drink was at the level of 90.15 mg/100 mL, and the dominant components were isoflavones (daidzein, glicitin, daidzin, genistin, glycitein, and genistein), which are characteristic of soybeans [32]. The presence of flavanones (naringenin and hesperidin) was also determined in the soy drink, which were not present in oat products (Table 4, Table S1 in Supplementary Materials). The addition of fruit powders to soy drink increased the content of polyphenols in the beverages. After adding V1 powder, the content of polyphenolic compounds increased to 126.49 mg/100 mL. As in the case of oat beverage, this matrix introduced compounds from the flavan-3-ols and procyanidins, phenolic acids, flavonols, and dihydrochalcones, derived from apples, into the solution.

After adding V2 powder, the content of polyphenolic compounds increased to 125.44 mg/100 mL. Interestingly, in the case of both soy beverages with the addition of V2 and V1 powder, no anthocyanins were detected. This fact is probably related to the masking of their presence by other groups of polyphenolic compounds, in which soy beverage is rich.

Changes in the polyphenolic composition of the plant-based beverages fortified with plant powders may have influenced the observed differences in flavor intensity and complexity, as well as for health-linked properties. Polyphenols, including phenolic acids, flavan-3-ols, and flavonols, are known to affect key sensory attributes such as bitterness, sourness, and astringency through both direct taste receptor activation and interactions with other food components. Phenolic acids, such as vanillic and ferulic acid, contribute to sour and bitter taste perception, respectively [33]. These effects are primarily due to their ability to precipitate salivary proteins, leading to tactile sensations such as dryness or roughness in the mouth [34]. Hence, an increase in phenolic acid content in the beverages could have amplified sour, bitter, and astringent notes, particularly in formulations where these compounds are abundant. Similarly, flavan-3-ols, especially in the form of proanthocyanidins, are strongly linked to astringency, which is intensified with higher degrees of polymerization due to stronger interactions with salivary proteins [34]. Flavonols, although less extensively studied in terms of sensory perception, also contribute to bitterness and may modulate flavor by interacting with both taste receptors and other polyphenols.

Beyond their sensory impact, phenolic acids, flavan-3-ols, and flavonols are also recognized for their beneficial health properties. These compounds exhibit strong antioxidant capacity and have been associated with reduced risk of chronic diseases such as cardiovascular disorders, certain cancers, and metabolic syndrome. Flavan-3-ols, in particular, have shown potential in improving vascular function and lowering blood pressure, while flavonols like quercetin may contribute to anti-inflammatory and immune-modulating effects [35,36].

### 3.4. Electronic Nose and Tongue

The electronic nose (e-nose) and electronic tongue (e-tongue) are analytical devices that simulate the function of the human nose and tongue, respectively, by using an array of gas or chemical sensors. These artificial sensing systems are primarily designed to assess food quality more quickly, consistently, and cost-effectively than traditional analytical methods such as GC-MS or HPLC. By using pattern recognition methods such as principal component analysis (PCA), neural networks (ANN), or support vector machines (SVM), these systems allow for effective classification and discrimination of samples [37].

#### 3.4.1. Electronic Nose

**Figure 2:**
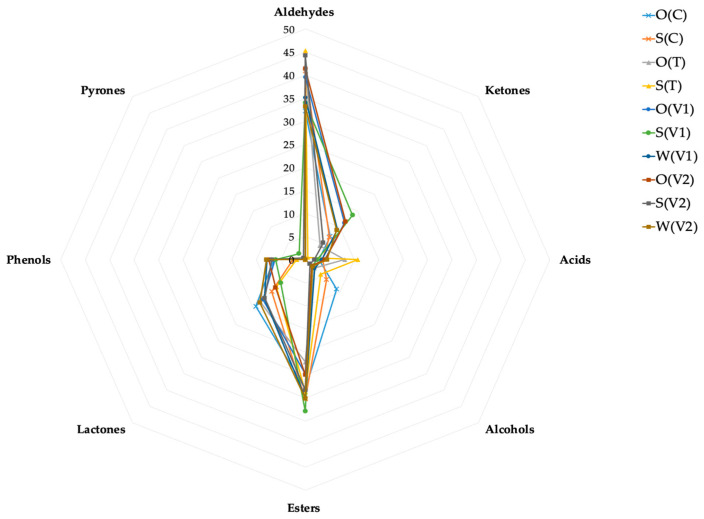
Radar chart showing the profiles of individual groups of aromatic compounds in the analyzed beverage samples; O(C)—control oat drink, S(C)—control soy drink, O(T)—trehalose in oat drink, S(T)—trehalose in soy drink, O(V1)—variant 1 in oat drink, S(V1)—variant 1 in soy drink, W(V1)—variant 1 in water, O(V2)—variant 2 in oat drink, S(V2)—variant 2 in soy drink, W(V2)—variant 2 in water.

The electronic nose analysis provided detailed insights into the volatile profiles of the tested plant-based beverages. The system identified key aroma compound classes (Figure 2), like aldehydes, ketones, acids, alcohols, esters, lactones, phenols, and pyrones, most of which contributed to desirable sensory notes. Compounds such as acetaldehyde and ethyl formate were associated with more intense, sharp aroma impressions.

The results indicated that most samples were dominated by compounds typically associated with pleasant aroma notes (Figure 2; Table S2). Only acetaldehyde and ethyl formate may contribute to the perception of sharpness in the aroma of the product. The highest concentrations of acetaldehyde were found in the control sample S(C) and in beverages based on oat, i.e., O(V1), O(V2), and one in soy S(V2) (Table S2 in Supplementary Materials). Ethyl formate reached its highest levels in the control sample S(C) as well as in the S(T), S(V2), and S(V1) beverages. In the S(T) sample, the 3-methylbutanoic acid, characterized by a sour, cheesy, rancid, and sweet aroma, was also identified, along with octyl isobutyrate, known for its waxy scent.

Oat- and soy-based beverages exhibited distinct volatile profiles, with 2-methylpropanal identified as the dominant compound across all samples. In oat-based drinks (e.g., control, O(T), O(V1), O(V2)) and water-based beverages (W(V1), W(V2)), key aroma compounds included 2-methylpropanal, furfural, butyl butanoate, 4-ethylguaiacol, and δ-decalactone, contributing fruity, sweet, and nutty notes. Soy-based samples (e.g., S(C), S(T), S(V1), S(V2)) were characterized by 2-methylpropanal, furfural, 3-heptanone, and ethyl heptanoate, with additional sweet and roasted aroma nuances. These results are consistent with the observations of Rabehi et al. [38], who emphasize that e-noses easily differentiate matrices of similar composition by analyzing the groups of volatile aromatic compounds.

**Figure 3:**
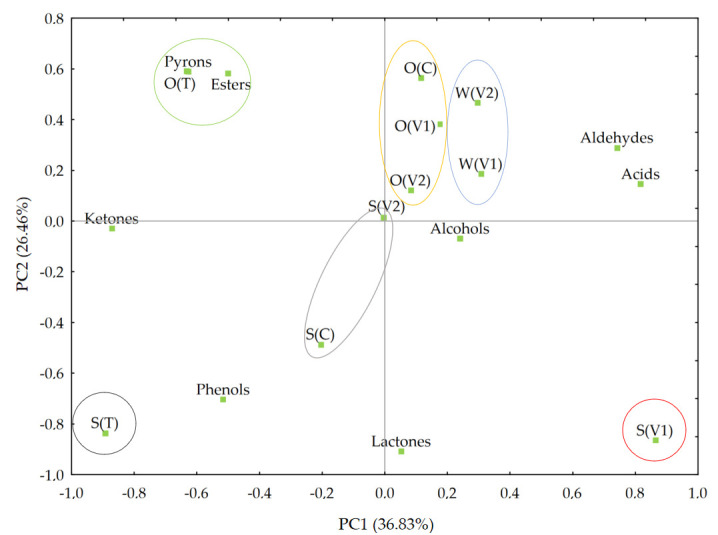
PCA chart showing the relationships between parameters related to the content of individual aromatic compounds in the analyzed beverage samples; O(C)—control oat drink, S(C)—control soy drink, O(T) trehalose in oat drink, S(T)—trehalose in soy drink, O(V1)—variant 1 in oat drink, S(V1)—variant 1 in soy drink, W(V1)—variant 1 in water, O(V2)—variant 2 in oat drink, S(V2)—variant 2 in soy drink, W(V2)—variant 2 in water.

Based on principal component analysis (PCA), six groups of samples were identified, each characterized by distinct aroma profiles and similar taste attributes within the group (Figure 3).

Samples such as S(T), O(T), and S(V1) differed significantly from the others, suggesting that the applied additives—such as carriers (e.g., trehalose) or specific matrices (e.g., soy, oat)—may have influenced the unique distribution of aroma compounds. Additionally, mutual interactions between these components could have contributed to changes in the composition and perception of volatile compounds. The second group was composed of S(C) and S(V2), which are likely to have a similar share of aldehydes and esters, which emphasizes the importance of the base composition (soy) in aroma modulation.

The greatest similarity in aroma compound profiles was observed among samples based on oat: O(C), O(V1), and O(V2). In these beverages, the dominant aroma compound was 2-methylpropanal, which imparts a fruity and malty scent. Another group composed of samples W(V1) and W(V2) showed very similar levels of furfural, associated with almond-like and sweet aroma notes. This indicated the simplification and unification of the aroma profile as a result of dissolution in water. Moreover, samples O(C), O(V2), O(V1), W(V1), and W(V2) were all characterized by a high content of butyl butanoate, a compound responsible for fresh, sweet, and fruity aroma impressions. These findings suggest that plant-based powders, due to their complex composition, should be evaluated within the final product matrix, as their interaction with other ingredients may either enhance or suppress specific sensory attributes depending on the formulation context. In this regard, the use of an electronic nose can serve as a valuable tool to support such assessments at an early stage by providing objective, reproducible data on aroma compound profiles and their variations across different matrices.

Electronic noses are widely used for monitoring fermentation processes, assessing meat freshness, and authenticating food products [39]. Their key advantage over traditional sensory panels lies in the reduction of subjective assessments and improved repeatability of results. However, similar to the human nose—which, despite its remarkable ability to distinguish a vast number of odors, is influenced by various factors [40]—the performance of e-nose systems can be affected by certain external conditions. These include environmental variables such as temperature and humidity, the need for consistent sample preparation, and the importance of using sufficiently large datasets to ensure accurate classification [41]. These aspects should be carefully considered during system design and application.

To the best of our knowledge, this study presents the first application of an electronic nose for profiling volatile compounds in soy- and oat-based beverages enriched with plant-derived powders. The findings highlight the potential of this technology as a practical tool for guiding formulation strategies that consider both functional and sensory quality. The e-nose enabled effective differentiation of complex aroma profiles, offering an objective method to support early-stage product development. As noted in previous studies [42], electronic noses are particularly suited to detecting subtle differences in aroma composition and can be further employed for quality monitoring and production standardization. Importantly, future research should incorporate sensory testing to evaluate consumer perception and confirm the sensory relevance of the observed aroma differences.

#### 3.4.2. Electronic Tongue

This study used the electronic tongue, a chemical sensor system that imitates the functioning of the human taste organ, to assess the taste profile of oat- and soy-based beverages and variants containing trehalose and V1 and V2 powders. The analysis included seven main taste attributes: sourness, metallicity, saltiness, hot spiciness, umami, sweetness, and bitterness.

**Figure 4:**
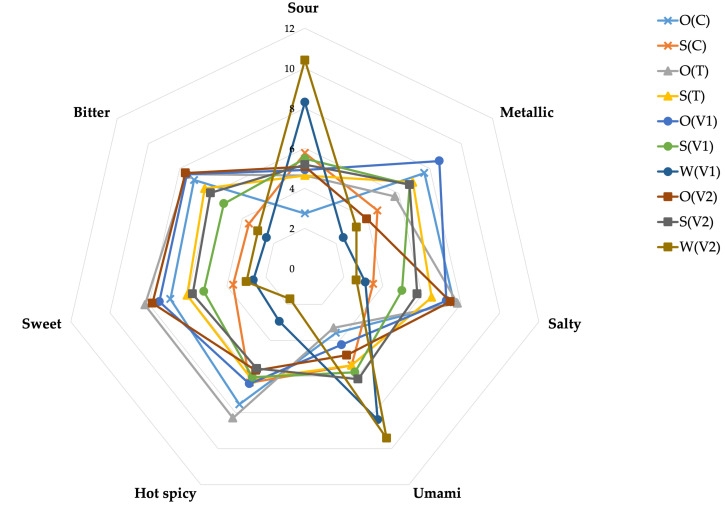
Radar chart showing the intensity of taste attributes in analyzed samples: sour, metallic, salty, hot spicy, umami, sweet, and bitter; O(C)—control oat drink, S(C)—control soy drink, O(T) trehalose in oat drink, S(T)—trehalose in soy drink, O(V1)—variant 1 in oat drink, S(V1)—variant 1 in soy drink, W(V1)—variant 1 in water, O(V2)—variant 2 in oat drink, S(V2)—variant 2 in soy drink, W(V2)—variant 2 in water.

The use of an electronic tongue in the evaluation of plant-based beverages enabled an effective analysis of their taste profile based on eight key sensory attributes. In this study, differences in taste profiles were observed between the variants, which were confirmed by both the radar plot (Figure 4; Table S3 in Supplementary Materials) and the principal component analysis (PCA). These results are supported by the literature, which emphasizes the high ability of e-tongues to distinguish samples with subtle sensory differences, especially in food products with a complex chemical matrix linked to application of different products [43,44]. In the control samples, the dominant taste in the soy beverage (without trehalose or powder additives; S(C)) was hot spicy, whereas in the oat beverage (O(C)), salty, metallic, and hot spicy notes were predominant. The addition of trehalose alone modified the taste profile, enhancing salty, sweet, bitter, and metallic notes in the soy beverage, while in the oat beverage, it intensified sour, sweet, and hot spicy sensations. In general, considering taste intensity, the sample O(V2) was distinguished by an intense bitter taste, whereas the W(V2) sample was characterized by pronounced sour and umami notes.

The analysis of the radar plot (Figure 4) showed a particular similarity between samples W(V1) and W(V2), which represented test variants dissolved in water. This indicates a significant effect of the matrix on taste perception—water-based beverages, devoid of complex flavor and aroma components present in soy or oat beverages, were characterized by a simplified and mutually similar sensory profile. Similar conclusions were drawn from studies on e-tongue analysis of wines and milk, where the type of medium modified the expression of key flavor attributes [43,45]. E-tongue systems are a valuable tool in food quality research, offering fast, repeatable, and bias-free results compared to traditional tasting panels [46]. The limitations of these systems include susceptibility to interference resulting from the complex sample matrix and the short life of the sensory materials, which requires frequent calibration of the devices and careful supervision of the analytical process [47].

**Figure 5:**
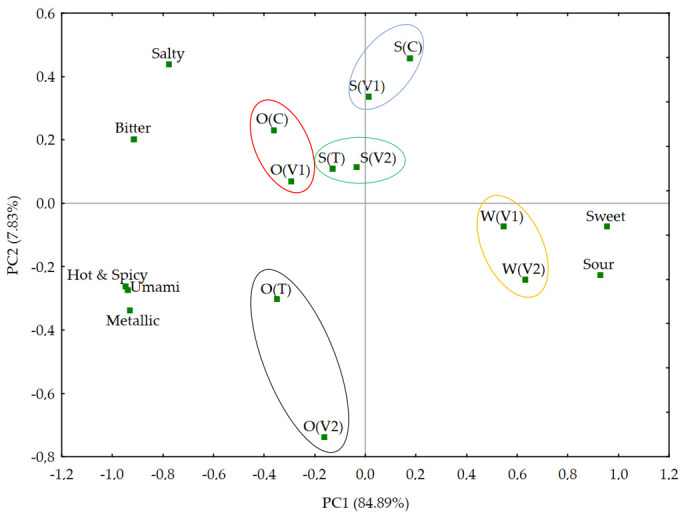
PCA chart showing the relationships between parameters related to the intensity of individual taste attributes in the analyzed beverage samples; O(C)—control oat drink, S(C)—control soy drink, O(T) trehalose in oat drink, S(T)—trehalose in soy drink, O(V1)—variant 1 in oat drink, S(V1)—variant 1 in soy drink, W(V1)—variant 1 in water, O(V2)—variant 2 in oat drink, S(V2)—variant 2 in soy drink, W(V2)—variant 2 in water.

Further PCA analysis (Figure 5) allowed the extraction of five clusters of samples with a similar taste profile, which confirms the effectiveness of this method in the sensory classification of beverages. It is worth noting that the first two principal components (PC1 and PC2) explained over 92% of the variance in total, which indicates a high level of data consistency and their usefulness in the sensory differentiation process. As emphasized by Pérez-Ràfols et al. [44], PCA is one of the most commonly used tools in the analysis of data generated by e-tongues and allows for effective dimensionality reduction while maintaining the ability to detect subtle differences between samples.

Based on PCA, five taste clusters can be distinguished: O(C) and O(V1)—oat beverage and variant 1 in the same medium—similarity suggests a small effect of variant 1 modification on taste in the context of oats; S(T) and S(V2)—clear similarity, which may result from the synergy between trehalose and variant 2 components in the soy medium; S(V1) and S(C)—also variant 1 and control in the soy beverage—indicates a similar basic taste; W(V1) and W(V2)—both variants in water—similarity stronger than any other, confirming the dominant effect of the medium; O(T) and O(V2)—variant 2 and trehalose in oat drink, which may indicate a common sweet or umami profile.

Of particular interest are the observations regarding the pairs of samples S(T) and S(V1) and O(T) and O(V2), which showed high consistency of taste profiles. The convergence of S(T) and S(V2) may result from the similar effect of trehalose and variant 2 components in the soy matrix. Trehalose is known for its taste-modifying properties—it can mask undesirable sensations, such as bitterness, while enhancing the perception of sweetness [48]. In the context of the presented results, this suggests a synergistic effect of the ingredients present in variant 2 and trehalose on sensory expression in soy beverages. In turn, the similarity of O(T) and O(V2), both containing additives in the oat drink, may indicate a high interaction of sweetening or functional ingredients with the oat matrix, which leads to taste convergence. The literature indicates that different matrices—water, protein, lipid—may expose the taste components contained in the additives to a different extent, which leads to different sensory receptions even with an identical set of additives [49].

**Figure 6:**
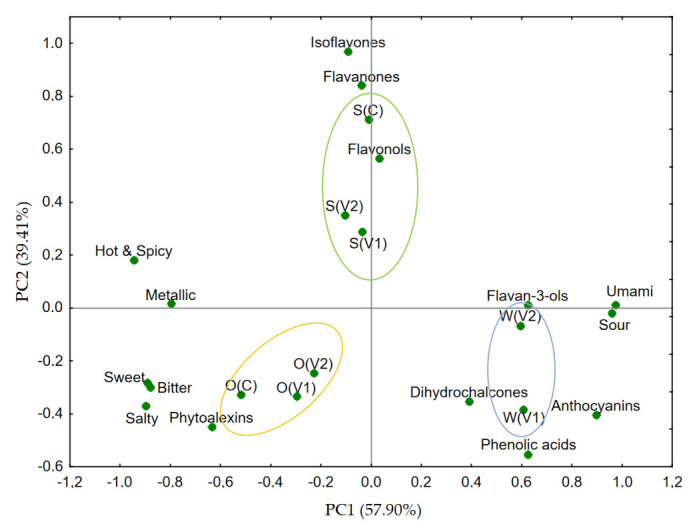
PCA bi-plot showing the relationships between parameters related to taste and phenolic compound groups; O(C)—control oat drink, S(C)—control soy drink, O(T) trehalose in oat drink, S(T)—trehalose in soy drink, O(V1)—variant 1 in oat drink, S(V1)—variant 1 in soy drink, W(V1)—variant 1 in water, O(V2)—variant 2 in oat drink, S(V2)—variant 2 in soy drink, W(V2)—variant 2 in water.

In order to determine the effect of polyphenolic compounds on the taste of the plant-based beverages, a PCA analysis was performed between the taste parameters and the individual groups of polyphenolic compounds present in the samples (Figure 6). The PC1 and PC2 axes explained, in total, 97.3% of the data variance.

After the analysis, four main clusters were distinguished: (1) polyphenols related to bitterness and astringency—in the lower left quadrant of the graph, there are tastes negatively correlated with the PC1 axis (bitter, salty, sweet), as well as compounds from the phytoalexins group. This indicated that phytoalexins are strongly correlated with bitterness and saltiness, which is consistent with literature reports on their interactions with T2R receptors [50,51]. The bitter taste of these compounds may reduce the sensory acceptance of food; (2) polyphenols causing astringency and umami—in the lower right quadrant of the PCA, flavan-3-ols (e.g., catechins), dihydrochalcones, anthocyanins, and phenolic acids can be distinguished near the umami and sour tastes. Flavanols and anthocyanins are strongly associated with astringency, which results from their ability to bind to salivary proteins and induce an ‘astringent’ effect. At the same time, these compounds can modulate the umami taste by affecting taste receptors in the mouth and gastrointestinal tract [52]; (3) polyphenols with sour and spicy potential—in the upper right quadrant, flavanones, isoflavones, and flavonols can be observed. These compounds are mainly associated with the perception of S(V1), S(V2), i.e., soy beverage. These groups of polyphenolic compounds are less bitter but more related to complex, sharp, and spicy notes (e.g., hot spicy and metallic) and possible influence on retronasal aromas [53,54]; (4) polyphenols and aromatic-flavor perception—groups located closer to the center of the graph, e.g., flavonols and the drink S(V2), characterized by a high content of these compounds. Polyphenols from the flavonols group can have a neutral or moderate effect on sensory perception, depending on the food matrix and concentration.

The PCA analysis confirmed that polyphenols such as flavanols, phytoalexins, and dihydrochalcones evoke taste sensations, such as bitterness, astringency, and sourness, while flavonols and isoflavones show more complex, often retronasal effects on taste and aroma. Targeted composition of the polyphenol profile can enhance or mask undesirable sensory characteristics of food products.

In the context of nutrition and food technology, polyphenols are known primarily for their health-related properties, as well as taste regulatory abilities, by causing bitterness and astringency [52,55]. From a sensory point of view, polyphenols interact with taste receptors in the oral cavity and with extraintestinal receptors, including in the gastrointestinal tract. These activities have a significant impact on the perception of bitterness and astringency, which in turn can affect consumer preferences, sensory acceptability of products, and the secretion of gut hormones that affect satiety and metabolism [50,51]. Polyphenols interact, among others, with salivary proteins, forming complexes that cause astringency and a flushing sensation, which can reduce the lubricating effect of saliva in the oral cavity.

In the current study, soy beverages (S(C), S(V1), S(V2)), characterized by the highest total polyphenols content (up to 126.49 mg/100 mL) (Table 4), showed the strongest correlation with flavanones, flavonols, and isoflavones, which were clearly associated with bitterness, metallic, and hot and spicy attributes in the PCA bi-plot (Figure 6). This supports the role of flavonoids in modulating these sensory qualities. Bitterness is produced by binding to T2R receptors, responsible for detecting bitter compounds such as catechins or procyanidins [50,51,53,54]. This is reflected in the S(V1) sample, where the presence of flavan-3-ols (33.22 mg/100 mL) (Table 4), particularly epicatechin and procyanidins, corresponded with strong umami and bitterness perception (Figure 6). Phenolic acids, such as chlorogenic, ferulic, or caffeic acid, mainly affect the aroma and flavor profile of products through reactions with volatile aromatic compounds and proteins. They can impart a slightly bitter taste, and their presence also affects the durability and stability of aromas [54]. In the oat variant O(V2), which showed a relatively high content of caffeoylhexose (12.74 mg/100 mL) (Table 4) and caffeic acid derivatives, a moderate association with sweet, bitter, and salty sensations was observed (Figure 6). Catechins and their oligomeric forms—procyanidins—exhibit strong astringency and bitterness properties. Their effect is strongly dependent on the degree of polymerization; monomeric catechins are more bitter, while oligomers exhibit more pronounced astringency [53]. This trend was evident in soy samples containing higher concentrations of B-type procyanidin oligomers and epicatechin, which aligned with astringent and bitter profiles (Figure 6). In the case of flavonols, such as quercetin or kaempferol, they exhibit moderate bitterness and a stabilizing effect on dyes and aromas. The presence of flavonols in soy-based beverages (S), particularly quercetin 3-*O*-glucoside and 3-*O*-xyloside, was strongly correlated with the metallic and hot sensations in PCA space (Figure 6). Flavanones, on the other hand, are responsible for the characteristic bitterness of these compounds. Soy drinks contained significant amounts of hesperidin and naringenin, especially in the control sample S(C), which corresponds with the sensory profile perceived. Another group of compounds present in the tested beverages are isoflavones, such as genistein, daidzein, and glycitein, characteristic of legumes. Their activation of bitter taste receptors aligns with the intense bitter and metallic sensation observed for soy variants, further confirmed by their distinct positioning in PCA near isoflavones (Figure 6). Phytoalexins (like avenanthramides), present mainly in oat drinks, correlated with bitter and salty notes in the PCA and were notably abundant in O(V1) and O(V2), supporting their potential influence on sensory profile—despite a limited focus in the literature to date [50].

While instrumental analysis offers precision and objectivity in sensory evaluation, it faces limitations when compared to human sensory evaluation. Instruments such as GC-MS and electronic noses or tongues are capable of accurately detecting and quantifying individual compounds; however, they fall short in replicating the holistic sensory experience perceived by humans [56]. This is because human sensory systems naturally integrate complex mixtures of stimuli without isolating individual components, relying on both peripheral sensitivity and central cognitive interpretation [57].

Electronic sensing systems attempt to mimic human taste and smell perception but often struggle with the stereoselectivity and nuanced complexity of odor and flavor profiles [58]. Moreover, instruments lack the ability to apply familiar descriptors or adapt to sensory context in the way trained panels can. Despite their cost and logistical limitations, human panels remain essential for tasks such as product taste design, adjustment, and quality control, offering qualitative insights that cannot be fully replicated by machines [56].

In conclusion, while instrumental methods are valuable for quantitative assessment, they must be seen as complementary rather than substitutive to human sensory analysis. The challenge of aligning instrumental outputs with subjective human experience persists, underscoring the irreplaceable role of trained panels in capturing the full sensory character of food and beverage products.

### 3.5. Processing Contaminants Contents

The powders were analyzed for processing contaminants to ensure the safety and compliance of the final products. The evaluation focused on the presence of heat-induced contaminants, including 5-hydroxymethyl-*L*-furfural (HMF), acrylamide, and furans, which can form during thermal processing, as these compounds may negatively impact product quality and consumer health. The analysis aimed to verify that contaminant levels remained within acceptable safety limits, ensuring the powders’ suitability for food applications. In the analyzed samples, all listed compounds in V1 and V2 powders were assessed using HPLC and GC methods. Their concentrations were found to be below the detection limits; therefore, these products can be considered safe for consumption in this regard.

## 4. Conclusions

The compositions of the plant-derived powders used in this study were developed using a New Product Development (NPD) approach, allowing for their effective incorporation as fortifying agents in popular plant-based beverages currently available on the market. These powders, obtained from popular fruits and herbs as well as fruits and vegetables with limited intrinsic sensory appeal, provide valuable phytochemical components that not only enrich the chemical profile of the final product but also modulate its sensory characteristics.

Physicochemical analyses confirmed the powders’ technological suitability as functional additives. In particular, their polyphenol content contributed to the functional value of the soy- and oat-based beverages, introducing compounds with proven health-promoting potential. Importantly, no process contaminants were detected in the analyzed formulations, further supporting the safety of these ingredients for food applications.

This study demonstrated, for the first time, the integrated application of an electronic nose and electronic tongue to characterize the volatile and taste-active profiles of fortified soy- and oat-based drinks. The electronic nose provided reproducible aroma differentiation, offering robust, objective support for sensory profiling during early-stage product development. The electronic tongue enabled reliable discrimination of taste profiles across formulations, revealing notable matrix-dependent effects, including a synergistic modulation of sweetness and bitterness in the presence of trehalose and specific powder combinations. 

Overall, the results confirm that fruit- and herb-based powders, particularly those derived from underutilized, bioactive-rich plant sources, hold strong potential as multifunctional ingredients. They contribute to both the nutritional enhancement and sensory improvement of plant-based beverages, supporting the development of innovative products that align with consumer expectations for health benefits, natural composition, and improved taste. Future work should include consumer-based sensory validation to bridge instrumental results with real-world preferences and acceptance.

## Figures and Tables

**Table 1:** The composition of blends used for powder preparation.

Raw Material	V1	V2
Apple juice	60%	–
Pear juice	–	50%
Japanese quince juice	–	25%
Rhubarb juice	21%	12.5%
Rosehip juice	11%	–
Lemon balm infusion	8%	12.5%
Brix (°) of the mixes	8.3 ± 0.1	6.8 ± 0.0

V1—powder compositions based on apple juice; V2—powder compositions based on pear juice.

**Table 2:** Physical properties of powders (V1 and V2) selected for beverage preparation.

Sample	Moisture Content (%)	Water Activity (—)	Color			WSI (%)
			L*	a*	b*	
V1	2.09 ± 0.06 a	0.1354 ± 0.004 a	57.95 ± 0.13 a	3.44 ± 0.02 a	12.26 ± 0.05 a	94.90 ± 0.99 a
V2	2.14 ± 0.01 a	0.1360 ± 0.001 a	58.69 ± 0.30 a	1.63 ± 0.02 b	9.20 ± 0.02 b	90.45 ± 0.21 b

WSI—water solubility index; V1—powder compositions based on apple juice; V2—powder compositions based on pear juice; a,b—different letters within the columns indicated statistically significant different values (Fisher’s LSD test, *p* < 0.05).

**Table 3:** Characteristic ions of bioactive compounds and their amounts (mg/g) in powders (V1 and V2) selected for beverage preparation.

MS [M − H]^−^/[M + H]^+^ (*m*/*z*) *	MS/MS Fragments (*m*/*z*) *	Tentative Identification	V1	V2
			(mg/g)	
Anthocyanins				
465.2344 ^+^	303.9030	Delphinidin-3-*O*-glucoside ^b^	0.01 ± 0.00 a	ND
611.7432 ^+^	303.7512	Delphinidin-3-*O*-rutinoside	0.06 ± 0.00 a	ND
449.0984 ^+^	287.0144	Cyanidin-3-*O*-glucoside ^b^	0.18 ± 0.01 a	0.15 ± 0.01 a
595.1774 ^+^	287.0503	Cyanidin 3-*O*-rutinoside	1.46 ± 0.01 a	1.11 ± 0.01 b
		Sum	1.71 ± 0.52 a	1.26 ± 0.40 b
Phenolic acids				
179.0811	165.0556	Caffeic acid	1.83 ± 0.02 a	1.81 ± 0.02 a
193.0602		Ferulic acid	1.18 ± 0.02 b	1.22 ± 0.01 a
341.0468	179.0148	Caffeoylhexose	1.75 ± 0.02 a	ND
190.9818	172.9777/128.9920	Quinic acid ^b^	1.36 ± 0.02 a	0.75 ± 0.01 b
365.0162	229.4809	Caffeoyl N-tryptophan	1.15 ± 0.02 a	ND
341.0509	179.0438	Caffeoylhexose	4.18 ± 0.09 a	1.91 ± 0.02 b
163.0380		*p*-Coumaric acid ^b^	0.28 ± 0.01 a	0.15 ± 0.01 b
515.1934	353.1473/191.0763	3,5-di-*O*-Caffeoylquinic acid	0.19 ± 0.01 a	ND
337.1073	173.9.9947	*trans*-4-*p*-Coumaroylquinic acid	4.33 ± 0.08 a	0.11 ± 0.01 b
387.0593	192.9996/134.5223	Ferulic truxilic acid	ND	3.08 ± 0.02 a
353.0306	191.0174	1-Caffeoylquinic acid ^b^	0.66 ± 0.01 a	ND
515.2367	353.0879/191.0554	3,4-di-*O*-Caffeoylquinic acid	ND	0.44 ± 0.01 a
341.1409	179.0677	Caffeoylhexose	ND	0.11 ± 0.00 a
353.0843	191.0960/179.0468	3-Caffeoylquinic acid ^b^	0.76 ± 0.01 b	1.03 ± 0.01 a
325.1466	191.0551/163.2115	*p*-Coumaroylhexose	ND	0.14 ± 0.00 a
499.1084	353.0879/337.0523/191.0548	*p*-Coumaroylcaffeoylquinic acid	ND	0.42 ± 0.01 a
337.1186	173.0446	*cis*-4-*p*-Coumaroylquinic acid	0.20 ± 0.00 a	ND
341.0627	179.0480	Caffeoylhexose	44.97 ± 0.24 a	ND
353.0283	191.0209/179.0011	5-Caffeoylquinic acid ^b^	0.92 ± 0.01 a	1.07 ± 0.01 a
353.0896	179.0426	*cis*-4-Caffeoylquinic acid	2.07 ± 0.02 a	0.57 ± 0.01 b
337.0972	173.0505/163.0469	*trans*-5-*p*-Coumaroylquinic acid	0.54 ± 0.01 a	ND
341.1122	191.0626	Caffeoylhexose	1.79 ± 0.01 a	ND
337.1011	173.0539	*cis*-5-*p*-Coumaroylquinic acid	3.66 ± 0.02 a	0.24 ± 0.01 b
691.1932	529.1307/515.1149/353.0918	Feruloyl-dicaffeoylquinic acid	ND	0.05 ± 0.00 a
597.2382	359.9091	Yunnaneic acid F	ND	4.12 ± 0.02 a
359.0802		Rosmarinic acid ^b^	3.53 ± 0.02 b	4.92 ± 0.03 a
249.0976		Unknown *p*-coumaric acid derivative	ND	0.20 ± 0.01 a
249.0972		Unknown *p*-coumaric acid derivative	ND	0.03 ± 0.00 a
337.0899	173.0387	3-*p*-Coumaroylquinic acid ^b^	ND	0.25 ± 0.02 a
499.1221	337.0878/163.0390	Caffeoyl-*p*-coumaroylquinic acid	ND	0.11 ± 0.01 a
451.0993	341.0660/179.0349/163.1194	Unknown caffeoylhexose derivative	ND	0.32 ± 0.01 a
367.1354	191.0541/174.9556	Feruloylquinic acid ^b^	ND	0.09 ± 0.00 a
		Sum	75.35 ± 0.21 a	23.14 ± 1.12 b
Flavan-3-ols and procyanidins				
575.1403	289.0717	A-type procyanidin dimer ^b^	ND	3.24 ± 0.02 a
577.1852	289.1011	B-type procyanidin dimer ^b^	ND	6.43 ± 0.02 a
289.0741		(+)-Catechin ^b^	4.39 ± 0.02 a	ND
577.1362	289.0237	B-type procyanidin dimer	0.55 ± 0.01 a	ND
865.1913	577.1187/289.0578	B-type procyanidin trimer	3.19 ± 0.01 b	22.14 ± 0.12 a
1153.1325	577.0475/289.0168	B-type procyanidin tetramer	0.74 ± 0.02 a	ND
577.2034	289.0967	B-type procyanidin dimer	ND	1.50 ± 0.01 a
577.1312	407.1258/287.0577	B-type procyanidin dimer	1.99 ± 0.02 b	3.87 ± 0.04 a
1153.3409	577.0475/289.0168	B-type procyanidin tetramer	ND	1.07 ± 0.01 a
1153.3531	289.1059	B-type procyanidin tetramer	ND	8.76 ± 0.03 a
575.1630	289.1061	A-type procyanidin dimer	ND	0.52 ± 0.01 a
865.1795	577.1144/287.0547	B-type procyanidin trimer	2.20 ± 0.02 a	0.39 ± 0.01 b
289.0808		(−)-Epicatechin ^b^	7.78 ± 0.15 a	7.76 ± 0.02 a
		Sum	20.84 ± 0.12 b	55.68 ± 0.58 a
Flavonols				
447.1028	285.0488	Kaempferol 3-*O*-galactoside ^b^	0.08 ± 0.00 a	ND
593.2101	285.0394	Kaempferol 3-*O*-rutinoside ^b^	ND	0.30 ± 0.00 a
609.1412	301.0356	Quercetin 3-*O*-rutinoside ^b^	1.31 ± 0.01 b	1.61 ± 0.02 a
463.0848	301.0355	Quercetin 3-*O*-galactoside ^b^	0.33 ± 0.01 a	0.30 ± 0.00 a
463.0940	301.0383	Quercetin 3-*O*-glucoside ^b^	0.58 ± 0.01 b	0.71 ± 0.02 a
593.1683	285.0768	Kaempferol 3-*O*-rutinoside	0.11 ± 0.01 a	ND
477.1170	315.0122	Isorhamnetin 3-*O*-galactodside	0.23 ± 0.01 a	ND
623.2241	315.0421	Isorhamnetin 3-*O*-rutinoside ^b^	0.10 ± 0.00 b	0.48 ± 0.02 a
447.0974	285.9372	Kaempferol 3-*O*-glucoside ^b^	0.60 ± 0.01 b	0.79 ± 0.02 a
595.1262	301.0258	Quercetin 3-*O*-glucosyl-xyloside	0.04 ± 0.00 a	ND
579.2061	285.0313	Kaempferol 3,7-di-*O*-rhamnoside	0.05 ± 0.00 a	ND
489.1042	285.0394	Kaempferol-3-*O*-6-acetylglucoside	ND	0.41 ± 0.00 a
433.1635	301.0355	Quercetin 3-*O*-arabinoside	0.37 ± 0.02 a	ND
519.1152	315.0327	Isorhamnetin-acylated hexoside	ND	2.83 ± 0.00 a
433.1628	301.0359	Quercetin 3-*O*-xyloside	0.15 ± 0.01 a	ND
519.1129	315.0512	Isorhamnetin-acylated hexoside	ND	1.00 ± 0.01 a
		Sum	3.95 ± 0.25 b	8.43 ± 0.09 a
Dihydrochalcones				
567.1042	273.0394	Phloretin 2′-*O*-xyloglucose	2.64 ± 0.03 a	ND
435.0762	273.0394	Phloretin 2′-*O*-glucose ^b^	1.94 ± 0.02 a	ND
		Sum	4.58 ± 0.08 a	ND
		TOTAL	106.43 ± 2.59 a	88.51 ± 1.26 b

* Experimental data; ^b^ Identified using corresponding authentic standards; Rt—retention time; ND—not detected; V1—powder compositions based on apple juice; V2—powder compositions based on pear juice. Means of three independent analyses ± standard deviation; a,b—values in the same rows followed by different letters are significantly different at *p* < 0.05 (Duncan’s test).

**Table 4:** Concentration of polyphenolic compounds (mg/100 mL) in oat and soy beverages and in beverages with plant powders.

	O(C)	O(V1)	O(V2)	S(C)	S(V1)	S(V2)
Phenolic acids	12.45 ± 0.43 b	29.01 ± 0.28 a	12.38 ± 1.01 b	6.67 ± 0.87 d	11.57 ± 0.98 bc	10.97 ± 0.52 c
Isoflavones	ND	ND	ND	65.62 ± 0.89 a	42.94 ± 1.02 b	44.17 ± 0.99 b
Anthocyanins	ND	0.10 ± 0.00 a	0.07 ± 0.00 a	ND	ND	ND
Flavan-3-ols and procyanidins	ND	16.50 ± 0.69 c	22.53 ± 1.02 b	3.94 ± 0.44 d	33.22 ± 0.32 a	30.69 ± 0.12 a
Phytoalexins	3.08 ± 0.09 a	0.61 ± 0.10 b	0.57 ± 0.12 b	ND	ND	ND
Flavonols	ND	1.63 ± 0.07 c	0.64 ± 0.05 d	5.92 ± 0.09 b	35.15 ± 0.12 a	37.94 ± 0.21 a
Flavanones	ND	ND	ND	7.93 ± 0.06 a	1.03 ± 0.05 b	1.65 ± 0.07 b
Dihydrochalcones	ND	2.43 ± 0.05 a	ND	0.07 ± 0.00 b	2.58 ± 0.03 a	0.02 ± 0.00 b
TOTAL	15.53 ± 2.46 e	50.28 ± 3.15 c	36.19 ± 2.87 d	90.15 ± 1.99 b	126.49 ± 1.67 a	125.44 ± 2.17 a

ND—not detected; O—oat; S—soy; C—control; V1—powder compositions based on apple juice; V2—powder compositions based on pear juice. Means of three independent analyses ± standard deviations; a,b,c,d,e—different letters in the same row show statistically significant differences between samples (Duncan’s test*, p* < 0.05).

## Data Availability

The original contributions presented in the study are included in the article/Supplementary Materials. Further inquiries can be directed to the corresponding author.

## Supplementary Materials

The following supporting information can be downloaded at: https://www.mdpi.com/article/10.3390/foods14152593/s1, Table S1: Content of polyphenolic compounds in plant-based beverages fortified with plant powders (mg/100 mL); Table S2: Volatile aroma compound profiles of the tested samples; Table S3: Results of the intensity assessment of sour, metallic, salty, umami, hot spicy, sweet, and bitter tastes in beverage samples using the electronic tongue system and Taste Screening tools; Figure S1: PCA chart showing the relationships between parameters related to the content of individual aromatic compounds in the analyzed beverage samples; O(C)—control oat drink, S(C)—control soy drink, O(T) trehalose in oat drink, trehalose in soy drink, O(V1)—variant 1 in oat drink, S(V1)—variant 1 in soy drink, W(V1)—variant 1 in water, O(V2)—variant 2 in oat drink, S(V2)—variant 2 in soy drink, W(V2)—variant 2 in water.
