# Supplementary material for: Physicochemical and Instrumental Flavor Analysis of Plant-Based Drinks with Plant Powder Additions
Source: Foods. 2025 Jul 24;14(15):2593. doi: 10.3390/foods14152593 (PMC12346121; doi:10.3390/foods14152593)
Supplement: Supplementary file 1 [file foods-14-02593-s001.zip › foods-3755247-supplementary.pdf]

Supplementary Materials

Table S1. Content of polyphenolic compounds in plant-based beverages fortified with plant powders (mg/100 mL).

MS [M-H]$^-$/[M+H]$^+$ (m/z)[a]	MS/MS fragments (m/z)[a]	Compound	mg/100mL					
			Oat			Soy		
			Oat beverage	V1	V2	Soy beverage	V1	V2
Phenolic acids								
179.0811	165.0556	Caffeic acid	ND	1.66 ± 0.01c	1.52 ± 0.02c	ND	3.06 ± 0.03a	2.68 ± 0.02b
193.0602		Ferulic acid	ND	1.81 ± 0.02b	2.01 ± 0.02a	0.12 ± 0.01c	0.02 ± 0.00c	0.04 ± 0.00c
341.0468	179.0148	Caffeoylhexose	ND	ND	ND	0.05 ± 0.00	0.26 ± 0.01	ND
365.0162	229.4809	Caffeoyl N-tryptophan	ND	1.03 ± 0.01a	ND	ND	ND	ND
341.0509	179.0438	Caffeoylhexose	ND	1.73 ± 0.02a	ND	ND	ND	ND
163.0380		*p*-Coumaric acid	ND	0.46 ± 0.02a	0.15 ± 0.01c	ND	0.21 ± 0.01b	0.10 ± 0.00d
337.1073	173.9.9947	*trans*-4-*p*-Coumaroylquinic acid	ND	1.63 ± 0.02a	0.54 ± 0.01b	ND	0.51 ± 0.01b	ND
387.0593	192.9996/134.5223	Ferulic truxilic acid	0.27 ± 0.01b	0.12 ± 0.00b	2.39 ± 0.01a	0.03 ± 0.00c	ND	0.13 ± 0.01b
341.0041	179.2749	Caffeoylhexose	7.46 ± 0.10a	1.04 ± 0.09c	1.94 ± 0.03b	ND	ND	ND
169.2318		Gallic acid	ND	ND	ND	0.89 ± 0.01a	0.09 ± 0.00b	0.10 ± 0.01b
353.0843	191.0960; 179.0468	3-Caffeoylquinic acid	0.21 ± 0.01c	0.99 ± 0.02b	2.00 ± 0.02a	ND	ND	ND
325.1466	191.0551, 163.2115	p-Coumaroylhexose	ND	ND	0.20 ± 0.00a	ND	ND	ND
137.2854		4-Hydroxybenzoic acid	ND	ND	ND	1.66 ± 0.02a	0.64 ± 0.01b	0.65 ± 0.01b
341.0627	179.0480	Caffeoylhexose	ND	12.74 ± 0.13a	ND	ND	1.41 ± 0.05b	ND
353.0896	179.0426	*cis*-4-Caffeoylquinic acid	ND	2.08 ± 0.02a	0.03 ± 0.00b	ND	ND	ND
341.1122	191.0626	Caffeoylhexose	2.41 ± 0.02a	2.51 ± 0.02a	0.49 ± 0.01c	0.12 ± 0.01d	1.53 ± 0.01b	0.05 ± 0.00d
337.1011	173.0539	*cis*-5-*p*-Coumaroylquinic acid	ND	0.47 ± 0.02bc	0.33 ± 0.02c	0.60 ± 0.03b	1.59 ± 0.03a	0.64 ± 0.0b1
325.1466	191.0551, 163.2115	*p*-Coumaroylhexose	0.99 ± 0.02a	0.28 ± 0.01b	0.28 ± 0.01b	ND	ND	ND
353.0283	191.0209/179.0011	5-Caffeoylquinic acid	1.11 ± 0.02a	0.16 ± 0.01b	0.16 ± 0.01b	ND	ND	ND
597.2382	359.9091	Yunnaneic acid F	ND	ND	ND	2.26 ± 0.03b	ND	3.29 ± 0.02a
359.0802		Rosmarinic acid	ND	0.30 ± 0.02d	0.34 ± 0.02d	0.94 ± 0.04c	2.25 ± 0.04b	3.29 ± 0.03a
		Sum	12.45 ± 0.43b	29.01 ± 0.28a	12.38 ± 1.01b	6.67 ± 0.87d	11.57 ± 0.98bc	10.97 ± 0.52c
Isoflavones								
253.1258		Daidzein	ND	ND	ND	9.66 ± 0.14a	5.14 ± 0.03b	5.95 ± 0.04b
445.1396	283.5711	Glicitin	ND	ND	ND	10.00 ± 0.09a	4.44 ± 0.02b	3.00 ± 0.02b
415.2596	253.0548	Daidzin	ND	ND	ND	5.00 ± 0.02a	2.14 ± 0.01b	2.45 ± 0.03b
431.1044	269.0599	Genistin	ND	ND	ND	20.12 ± 0.12a	14.91 ± 0.16b	15.81 ± 0.13b

283.0699		Glycitein		ND	ND	ND	10.14 ± 0.12a	8.16 ± 0.08b	9.04 ± 0.10ab
269.0125		Genistein		ND	ND	ND	10.70 ± 0.12a	8.15 ± 0.08b	7.92 ± 0.09b
			Sum	ND	ND	ND	65.62 ± 0.89a	42.94 ± 1.02b	44.17 ± 0.99b
Anthocyanins									
595.1774	287.0503	Cyanidin 3-*O*-rutinoside		ND	0.10 ± 0.00a	0.07 ± 0.00a	ND	ND	ND
			Sum	ND	0.10 ± 0.00a	0.07 ± 0.00a	ND	ND	ND
Flavan-3-ols and Procyanidins									
575.1403	289.0717	A-type procyanidin dimer		ND	ND	ND	0.08 ± 0.00b	ND	0.53 ± 0.01a
577.1852	289.1011	B-type procyanidin dimer		ND	ND	0.19 ± 0.01a	ND	ND	ND
289.0741		(+)-Catechin		ND	ND	ND	ND	9.87 ± 0.12a	ND
865.1913	577.1187, 289.0578	B-type procyanidin trimer		ND	ND	ND	0.05 ± 0.00c	1.01 ± 0.00b	2.15 ± 0.00a
577.2034	289.0967	B-type procyanidin dimer		ND	ND	7.32 ± 0.03a	ND	ND	ND
577.1312	407.1258, 287.0577	B-type procyanidin dimer		ND	ND	ND	ND	0.01 ± 0.00b	0.35 ± 0.00a
1153.3531289.1059		B-type procyanidin tetramer		ND	ND	ND	0.36 ± 0.01b	ND	6.63 ± 0.11a
865.1795	577.1144, 287.0547	B-type procyanidin trimer		ND	2.87 ± 0.02c	0.54 ± 0.01d	3.45 ± 0.03b	4.51 ± 0.02a	3.83 ± 0.02b
289.0808		(−)-Epicatechin		ND	13.63 ± 0.08c	14.48 ± 0.14b	ND	17.82 ± 0.13a	17.20 ± 0.10a
			Sum	ND	16.50 ± 0.69d	22.53 ± 1.02c	3.94 ± 0.44e	33.22 ± 0.32a	30.69 ± 0.12b
Phytoalexins									
229.8761	146.0879	Avenanthramide A		2.11 ± 0.02a	0.39 ± 0.01b	0.37 ± 0.01b	ND	ND	ND
329.0877	176.0589	Avenanthramide B		0.97 ± 0.02a	0.22 ± 0.03b	0.20 ± 0.02b	ND	ND	ND
			Sum	3.08 ± 0.09a	0.61 ± 0.10b	0.57 ± 0.12b	ND	ND	ND
Flavonols									
609.1412	301.0356	Quercetin 3-*O*-rutinoside		ND	ND	ND	5.78 ± 0.09b	6.24 ± 0.02a	6.49 ± 0.04a
463.0848	301.0355	Quercetin 3-*O*-galactoside		ND	0.57 ± 0.01a	0.25 ± 0.01b	ND	ND	ND
463.0940	301.0383	Quercetin 3-*O*-glucoside		ND	0.16 ± 0.01c	0.16 ± 0.01c	ND	26.8 ± 0.15b	29.58 ± 0.21a
593.1683	285.0768	Kaempferol 3-*O*-rutinoside		ND	0.74 ± 0.05a	ND	ND	ND	ND
447.0974	285.9372	Kaempferol 3-*O*-glucoside		ND	0.16 ± 0.01b	0.23 ± 0.02a	ND	ND	ND
433.1628	301.0359	Quercetin 3-*O*-xyloside		ND	ND	ND	0.14 ± 0.01c	2.11 ± 0.03a	1.87 ± 0.02b
519.1129	315.0512	Isorhamnetin-acylatedhexoside		ND	ND	ND	ND	ND	ND
			Sum	ND	1.63 ± 0.07c	0.64 ± 0.05d	5.92 ± 0.09b	35.15 ± 0.12a	37.94 ± 0.21a
Flavanones									
271.4589		Naringenin		ND	ND	ND	2.59 ± 0.02a	0.52 ± 0.02b	0.55 ± 0.03b
609.0589		Hesperidin		ND	ND	ND	5.34 ± 0.03a	0.51 ± 0.02c	1.10 ± 0.02b
			Sum	ND	ND	ND	7.93 ± 0.06a	1.03 ± 0.05c	1.65 ± 0.07b
Dihydrochalcones									
567.1042	273.0394	Phloretin 2′*O*-xyloglucose		ND	1.00 ± 0.01a	ND	ND	0.99 ± 0.01a	ND

435.0762	273.0394	Phloretin 2′-*O*-glucose		ND	1.43 ± 0.03a	ND	0.07 ± 0.00b	1.59 ± 0.01a	0.02 ± 0.00b
			Sum	ND	2.43 ± 0.05a	ND	0.07 ± 0.00b	2.58 ± 0.03a	0.02 ± 0.00b
			TOTAL	15.53 ± 2.46e	50.28 ± 3.15c	36.19 ± 2.87d	90.15 ± 1.99b	126.49 ± 1.67a	125.44 ± 2.17a

Means of three independent analyses ± standard deviation; Rt – retention time; ND – not detected; V1 - variant 1; V2 - variant 2; a,b,c... – different letters in the rows indicated a statistically significant differences (Duncan test, $p < 0.05$). [a]Experimental data

Table S2. Volatile aroma compound profiles of the tested samples.

	**O(C)**	**S(C)**	**O(T)**	**S(T)**	**O(V1)**	**S(V1)**	**W(V1)**	**O(V2)**	**S(V2)**	**W(V2)**
Acetaldehyde	ND	7.07 ± 0.04b	ND	ND	7.90 ± 0.04a	4.86 ± 0.01e	2.92 ± 0.01f	6.55 ± 0.03d	6.60 ± 0.03c	1.41 ± 0.01g
Ethyl formate	ND	12.75 ± 0.06b	ND	14.59 ± 0.07a	1.15 ± 0.01g	6.38 ± 0.02d	3.70 ± 0.02e	1.20 ± 0.01g	11.14 ± 0.06c	3.19 ± 0.02f
2-methylpropanal	22.73 ± 0.33d	29.41 ± 0.35b	25.17 ± 0.38c	31.01 ± 0.35a	18.83 ± 0.41f	10.84 ± 0.26h	15.98 ± 0.42g	19.94 ± 0.40e	22.51 ± 0.39d	15.81 ± 0.42g
Butane-2,3-dione	ND	ND	ND	ND	4.47 ± 0.02a	2.65 ± 0.01e	3.20 ± 0.02c	3.30 ± 0.02b	1.59 ± 0.01f	3.11 ± 0.02d
Butan-2-one	ND	ND	ND	ND	3.29 ± 0.02b	3.37 ± 0.01a	ND	2.29 ± 0.01c	0.73 ± 0.00d	ND
3-Methylbutanal	ND	1.70 ± 0.01b	1.27 ± 0.01d	9.59 ± 0.05a	1.23 ± 0.01e	0.94 ± 0.00f	1.35 ± 0.01c	1.26 ± 0.01d	0.68 ± 0.00g	1.32 ± 0.01c
Pentan-2-one	1.27 ± 0.01a	0.87 ± 0.00b	ND	0.66 ± 0.00c	ND	0.22 ± 0.00d	ND	ND	ND	ND
2-Methylpropanoic acid	ND	ND	3.88 ± 0.02a	ND	ND	ND	ND	1.76 ± 0.01b	ND	ND
Furfural	5.92 ± 0.03g	0.93 ± 0.00i	9.59 ± 0.05b	0.77 ± 0.00j	7.16 ± 0.04f	11.69 ± 0.03a	8.99 ± 0.05d	7.77 ± 0.04e	4.96 ± 0.02h	9.47 ± 0.05c
3-Methylbutanoic acid	ND	ND	ND	6.02 ± 0.03a	ND	1.08 ± 0.00b	ND	ND	ND	ND
3-Heptanone	3.97 ± 0.02e	5.85 ± 0.03bc	4.16 ± 0.02d	ND	3.40 ± 0.02f	6.85 ± 0.02a	5.87 ± 0.03b	5.84 ± 0.03bc	2.22 ± 0.01g	5.82 ± 0.03c
Benzaldehyde	3.90 ± 0.02f	0.64 ± 0.00i	5.27 ± 0.03a	2.76 ± 0.01g	3.97 ± 0.02e	4.90 ± 0.01b	5.30 ± 0.03a	4.20 ± 0.02d	2.12 ± 0.01h	4.68 ± 0.02c
Butyl butanoate	6.25 ± 0.03d	3.80 ± 0.02g	5.91 ± 0.03e	2.91 ± 0.01h	6.27 ± 0.03d	9.57 ± 0.03a	8.44 ± 0.04b	8.28 ± 0.04c	3.92 ± 0.02f	8.28 ± 0.04c
Octanal	ND	ND	ND	ND	ND	ND	ND	0.92 ± 0.00a	ND	ND
(Z)-2-octenal	ND	ND	ND	ND	ND	ND	ND	ND	6.74 ± 0.03a	ND
5-Ethyldihydro-2(3h)-furanone	4.84 ± 0.02b	3.08 ± 0.02i	4.31 ± 0.02d	4.70 ± 0.02c	3.70 ± 0.02f	4.18 ± 0.01e	3.12 ± 0.02h	3.27 ± 0.02g	5.83 ± 0.03a	3.25 ± 0.02g
2-Phenylethanol	1.89 ± 0.01c	5.92 ± 0.03a	ND	1.98 ± 0.01b	ND	0.96 ± 0.00d	ND	ND	ND	ND
ethyl heptanoate	3.19 ± 0.01f	2.10 ± 0.01g	4.01 ± 0.02c	ND	3.75 ± 0.02d	4.56 ± 0.01a	4.35 ± 0.02b	3.57 ± 0.02e	3.56 ± 0.02e	4.57 ± 0.02a
Maltol	ND	ND	ND	ND	ND	1.79 ± 0.01a	ND	ND	0.52 ± 0b	ND
(Z)-2-decenal	7.19 ± 0.03a	ND	2.53 ± 0.01c	2.35 ± 0.01d	1.87 ± 0.01f	0.98 ± 0.00i	2.63 ± 0.01b	1.85 ± 0.01g	1.29 ± 0.01h	2.06 ± 0.01e
4-Ethylguaiacol	6.50 ± 0.03e	2.46 ± 0.01h	7.82 ± 0.04a	1.73 ± 0.01i	6.15 ± 0.03f	5.92 ± 0.02g	7.67 ± 0.04b	7.33 ± 0.04c	6.76 ± 0.03d	7.81 ± 0.04a
Octyl isobutyrate	ND	1.89 ± 0.01b	ND	4.76 ± 0.02a	1.18 ± 0.01e	1.07 ± 0.00f	1.20 ± 0.01d	ND	1.20 ± 0.01d	1.24 ± 0.01c
Gamma-nonalactone	1.16 ± 0.01g	5.35 ± 0.03a	1.64 ± 0.01e	ND	2.04 ± 0.01d	0.45 ± 0.00i	2.36 ± 0.01c	1.03 ± 0.01h	1.45 ± 0.01f	2.60 ± 0.01b

Pentyl octanoate	5.53 ± 0.02a	0.68 ± 0.00i	4.89 ± 0.02d	1.13 ± 0.01h	4.51 ± 0.02f	4.45 ± 0.01g	5.03 ± 0.03c	4.46 ± 0.02g	4.66 ± 0.02e	5.38 ± 0.03b
Butanoic acid, 3-methyl	5.42 ± 0.02e	1.01 ± 0.01j	6.80 ± 0.03b	3.43 ± 0.02h	6.36 ± 0.03c	2.32 ± 0.01i	6.16 ± 0.03d	4.16 ± 0.02g	4.31 ± 0.02f	7.11 ± 0.04a
Butanoic acid	2.85 ± 0.01g	3.39 ± 0.02e	4.02 ± 0.02c	4.44 ± 0.02a	3.90 ± 0.02d	1.29 ± 0.00j	3.37 ± 0.02f	2.20 ± 0.01h	1.83 ± 0.01i	4.35 ± 0.02b
4-Undecanolide	2.10 ± 0.01d	3.00 ± 0.02a	1.71 ± 0.01f	2.62 ± 0.01b	1.23 ± 0.01j	2.42 ± 0.01c	1.60 ± 0.01g	1.79 ± 0.01e	1.47 ± 0.01i	1.56 ± 0.01h
Ethyl dodecanoate	3.50 ± 0.02a	1.32 ± 0.01b	ND	0.90 ± 0.00c	ND	0.58 ± 0.00d	ND	ND	ND	ND
Isopentyl salicylate	ND	1.33 ± 0.01c	ND	ND	ND	1.09 ± 0.00d	ND	4.57 ± 0.02a	ND	1.67 ± 0.01b
Benzophenone	1.99 ± 0.01a	ND	ND	ND	ND	0.23 ± 0.00c	ND	0 ± 0	0.54 ± 0.00b	ND
Benzyl benzoate	4.15 ± 0.02b	3.15 ± 0.02f	3.69 ± 0.02e	ND	4.19 ± 0.02a	1.43 ± 0.00i	3.78 ± 0.02d	2.46 ± 0.01g	2.22 ± 0.01h	3.90 ± 0.02c
Ethyl tetradecanoate	3.44 ± 0.01a	0.62 ± 0.00h	1.37 ± 0.01g	2.56 ± 0.01b	1.98 ± 0.01d	2.01 ± 0.01c	1.83 ± 0.01e	ND	0.58 ± 0.00i	1.42 ± 0.01f
Benzyl phenyl acetate	2.19 ± 0.01a	1.67 ± 0.01c	1.96 ± 0.01b	1.09 ± 0.01f	1.47 ± 0.01d	0.92 ± 0.00g	1.18 ± 0.01e	ND	0.58 ± 0.00h	ND

O(C) – control oat drink, S(C) – control soy drink, O(T) – trehalose in oat drink, S(T) – trehalose in soy drink, O(V1) – variant 1 in oat drink, S(V1) – variant 1 in soy drink, W(V1) – variant 1 in water, O(V2) – variant 2 in oat drink, S(V2) – variant 2 in soy drink, W(V2) – variant 2 in water; ND – not detected; a,b,c … – different letters in the rows indicated a statistically significant differences (Duncan test, $p < 0.05$).

Table S3. Results of the intensity assessment of sour, metallic, salty, umami, hot spicy, sweet, and bitter tastes in beverage samples using the electronic tongue system and Taste Screening tools

Sample	Sour	Metallic	Salty	Umami	Hot spicy	Sweet	Bitter
O(C)	2.74 ± 0.11g	7.64 ± 0.32b	7.64 ± 0.32ab	3.58 ± 0.15h	7.54 ± 0.32b	6.90 ± 0.29d	7.08 ± 0.29b
S(C)	5.76 ± 0.25c	4.64 ± 0.18e	3.50 ± 0.14g	5.38 ± 0.22e	6.34 ± 0.25cd	3.68 ± 0.15g	3.58 ± 0.15f
O(T)	4.64 ± 0.18f	5.76 ± 0.25d	7.82 ± 0.33a	3.30 ± 0.14h	8.30 ± 0.36a	8.20 ± 0.36a	7.54 ± 0.32a
S(T)	4.64 ± 0.18f	6.90 ± 0.29c	6.50 ± 0.29d	5.38 ± 0.22e	6.14 ± 0.25cd	6.04 ± 0.25e	6.40 ± 0.29c
O(V1)	4.92 ± 0.22ef	8.60 ± 0.36a	7.26 ± 0.32c	4.24 ± 0.18g	6.40 ± 0.29c	7.46 ± 0.32c	7.54 ± 0.32a
S(V1)	5.48 ± 0.22cd	6.70 ± 0.29c	4.98 ± 0.22f	5.76 ± 0.25d	6.04 ± 0.25d	5.18 ± 0.22f	5.18 ± 0.22e
W(V1)	8.30 ± 0.36b	2.46 ± 0.11h	3.10 ± 0.14h	8.40 ± 0.36b	2.94 ± 0.11f	2.64 ± 0.11i	2.46 ± 0.11h
O(V2)	5.08 ± 0.22e	3.96 ± 0.18f	7.46 ± 0.32bc	4.82 ± 0.22f	5.66 ± 0.25e	7.82 ± 0.33b	7.64 ± 0.32a
S(V2)	5.18 ± 0.22de	6.70 ± 0.29c	5.76 ± 0.25e	6.14 ± 0.25c	5.56 ± 0.25e	5.76 ± 0.25e	6.04 ± 0.25d
W(V2)	10.40 ± 0.43a	3.30 ± 0.14g	2.64 ± 0.11i	9.42 ± 0.40a	1.70 ± 0.07g	3.00 ± 0.14h	3.00 ± 0.14g

O(C) – control oat drink, S(C) – control soy drink, O(T) – trehalose in oat drink, S(T) – trehalose in soy drink, O(V1) – variant 1 in oat drink, S(V1) – variant 1 in soy drink, W(V1) – variant 1 in water, O(V2) – variant 2 in oat drink, S(V2) – variant 2 in soy drink, W(V2) – variant 2 in water; a,b,c... – different letters in the columns indicated a statistically significant differences (Duncan test, $p < 0.05$).

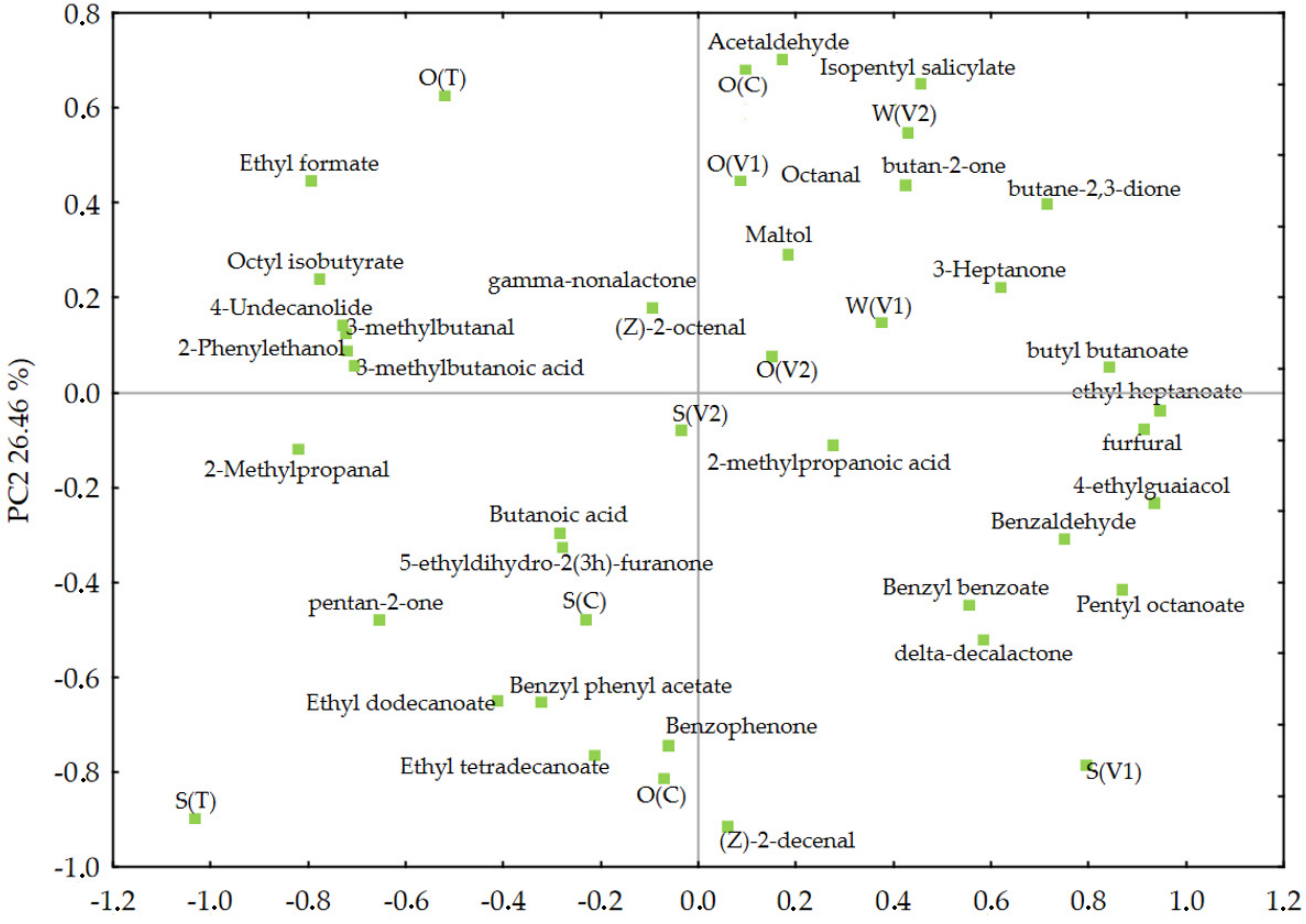

Figure S1. PCA chart showing the relationships between parameters related to the content of individual aromatic compounds in the analyzed beverage samples; O(C) – control oat drink, S(C) – control soy drink, O(T) trehalose in oat drink, trehalose in soy drink, O(V1) – variant 1 in oat drink, S(V1) – variant 1 in soy drink, W(V1) – variant 1 in water, O(V2) – variant 2 in oat drink, S(V2) – variant 2 in soy drink, W(V2) – variant 2 in water.
